# O8-6 Participation in physical education, extra-curricular activities, and community sports among Irish adolescents with and without functional difficulties

**DOI:** 10.1093/eurpub/ckac094.062

**Published:** 2022-08-29

**Authors:** Kwok Ng, Sarahjane Belton, Marie Murphy, Wesley O'Brian, Catherine Woods

**Affiliations:** 1 Department of Physical Education and Sport Sciences, University of Limerick, Limerick, Ireland; 2 School of Educational Sciences & Psychology, University of Eastern Finland, Joensuu, Finland; 3 School of Health & Human Performance, Dublin City University, Dublin, Ireland; 4 Ulster University Doctoral College, Ulster University, Belfast, United Kingdom; 5 School of Education, University College Cork, Cork, Ireland

**Keywords:** Disabilities, health promotion, adapted physical activity, surveillance, Ireland

## Abstract

**Background:**

Physical activity for adolescents with disabilities (AWD) are reported to have even greater health benefits than for adolescents without disabilities (AWoD). The settings for organised physical activity opportunities can include physical education, extra-curricular activities and community sport. Few studies have reported whether there are differences in participation in these settings between AWD and AWoD. The purpose of this study was to report differences in participation in organised physical activity between AWD and AWoD in Ireland.

**Methods:**

Data, were disaggregated by disabilities, from the Irish children sport participation and physical activity 2018 study; a national representative self-report survey. Adolescents selected sports and physical activities they took part in the last 12 months in physical education, extra-curricular activities, and community sports. The child functioning module was completed with data coded according to the Washington group on disability statistics criteria. Data were stratified by gender and school level, with average scores of the number of activities analysed by T-Tests with Hedge's g, and no participation by Chi-square test of independence.

**Results:**

The weighted sample included 6646 adolescents (53% female, 68% secondary level), of which 16% reported disabilities. Specific difficulties were sensory (4%), physical (1%), cognitive (7%), and behavioural (9%). More AWD reported they did not do any organised physical activities in all three settings (physical education, p = 0.029, extra-curriculum, p = >.001, community sport p = >.001) than AWoD. Adolescents with behavioural disabilities reported fewer types of physical education activities (males primary, p = .014, g =.31; secondary, p = .008, g = .24) and community sports (male primary, p = >.001, g = .49; female secondary, p = .027, g = .14) than adolescents without behavioural disabilities.

**Conclusions:**

Adolescents with behavioural difficulties were the largest disability group and reported fewer number of organised physical activities than AWoD; reinforcing actions are needed to increase perceived choice of activities. An alarming number of AWD reported no organised physical activities in all three settings. More thorough studies are required to investigate these reasons, and to provide support across settings, whereby differences in participation between adolescents with and without disabilities are not so profound.

